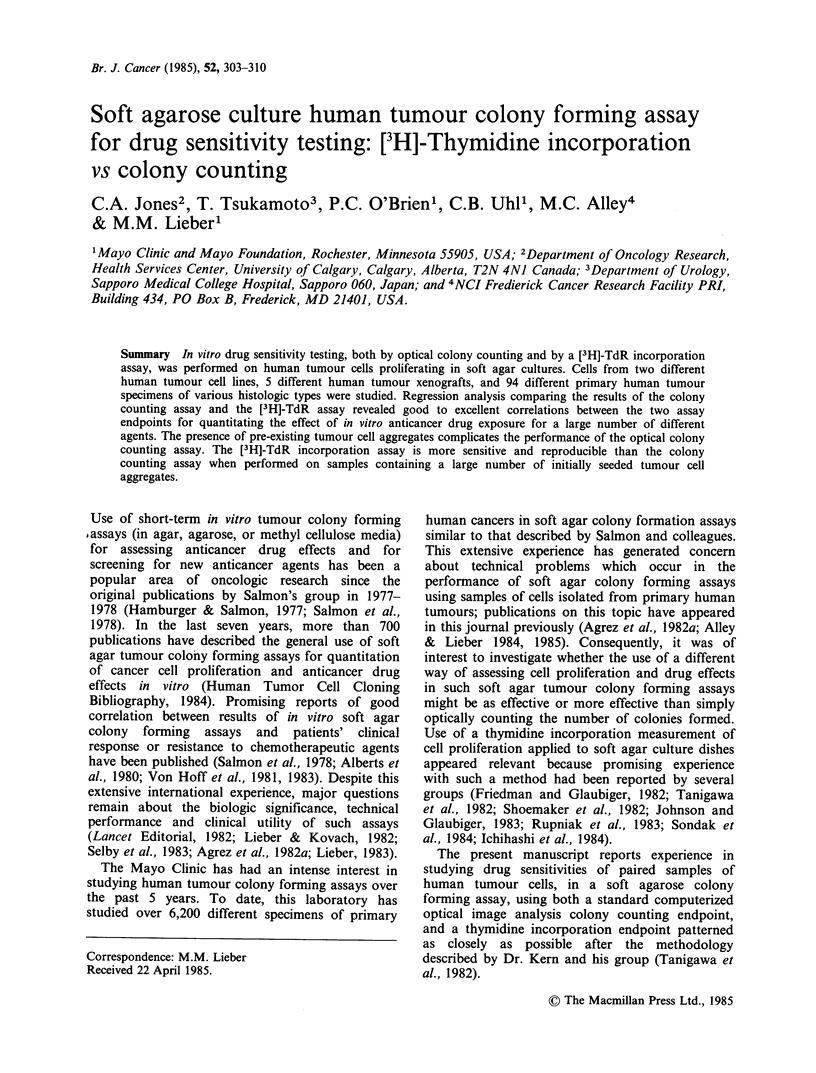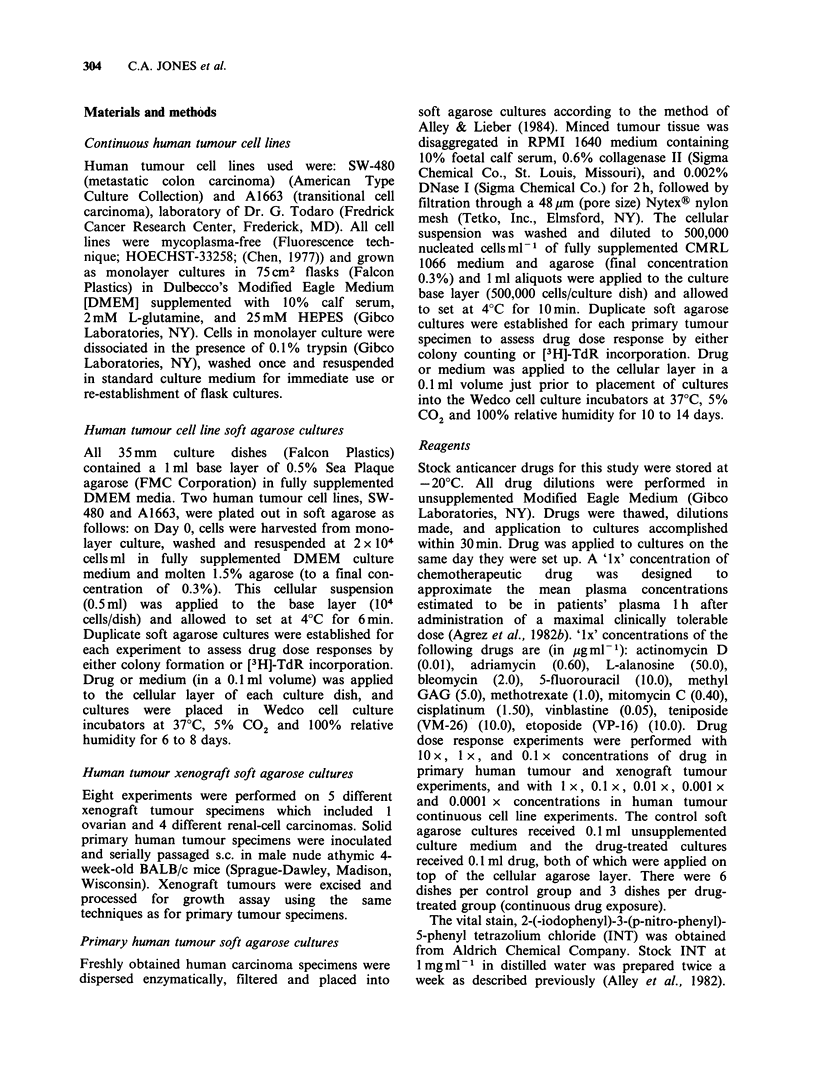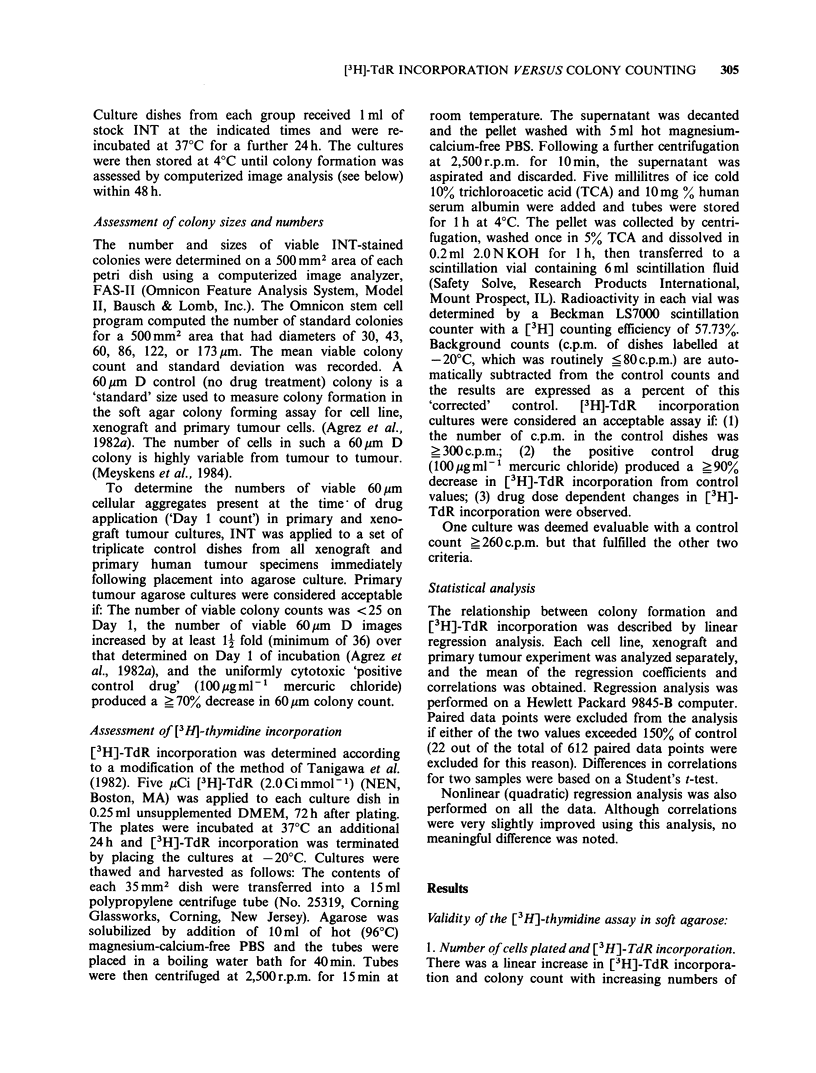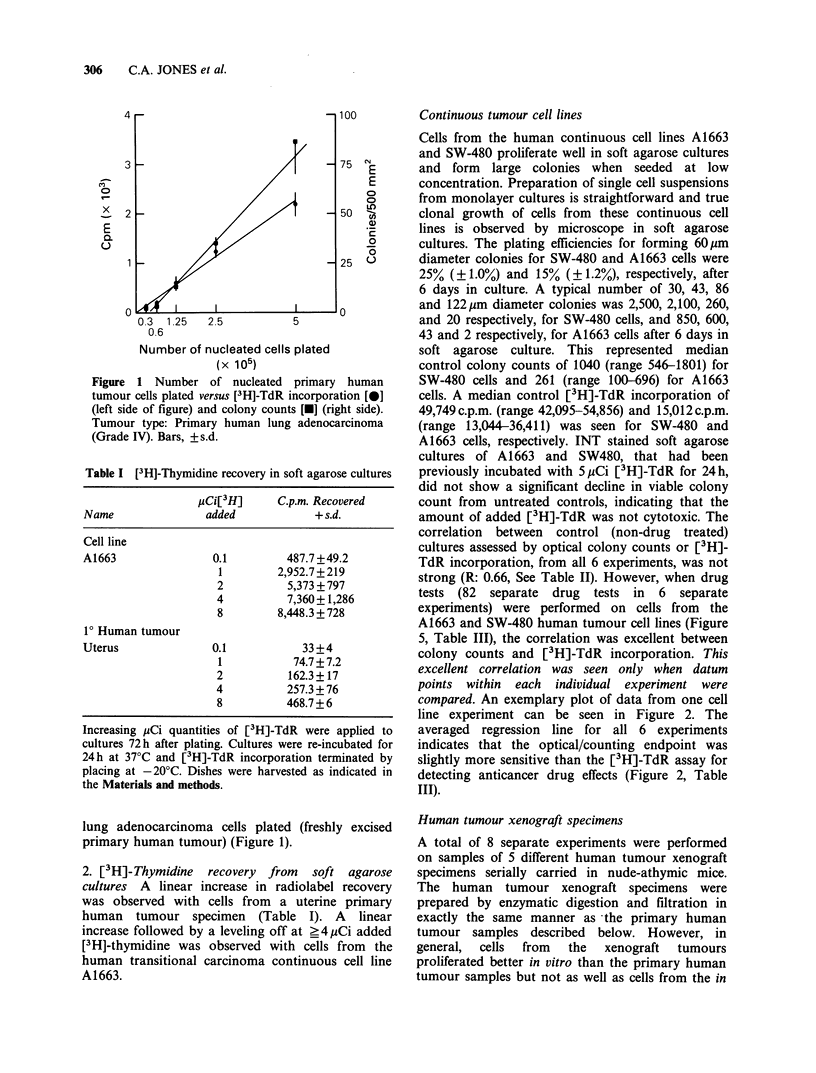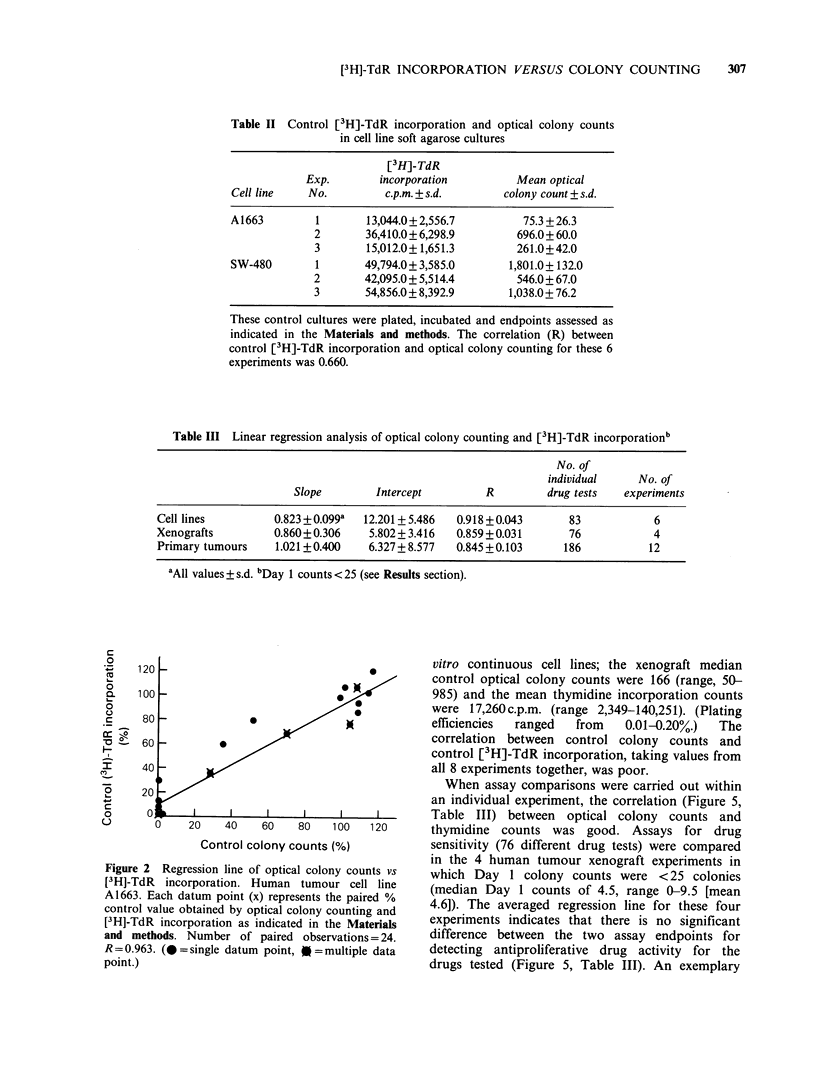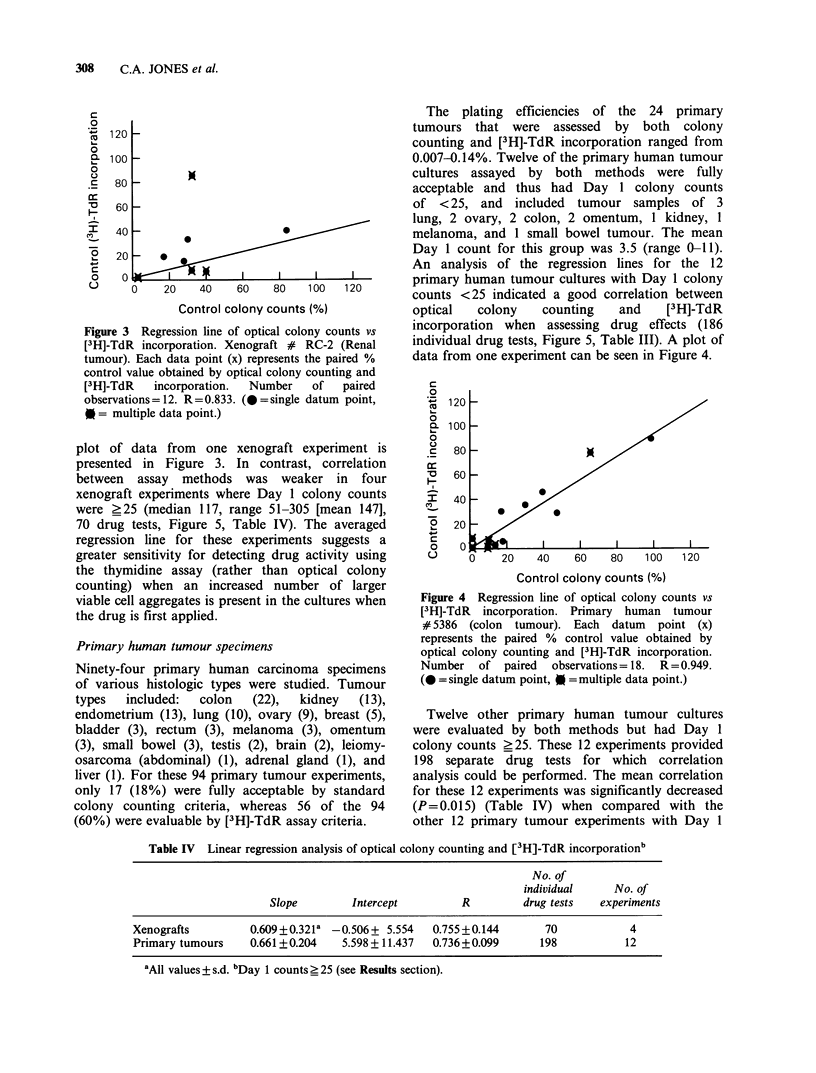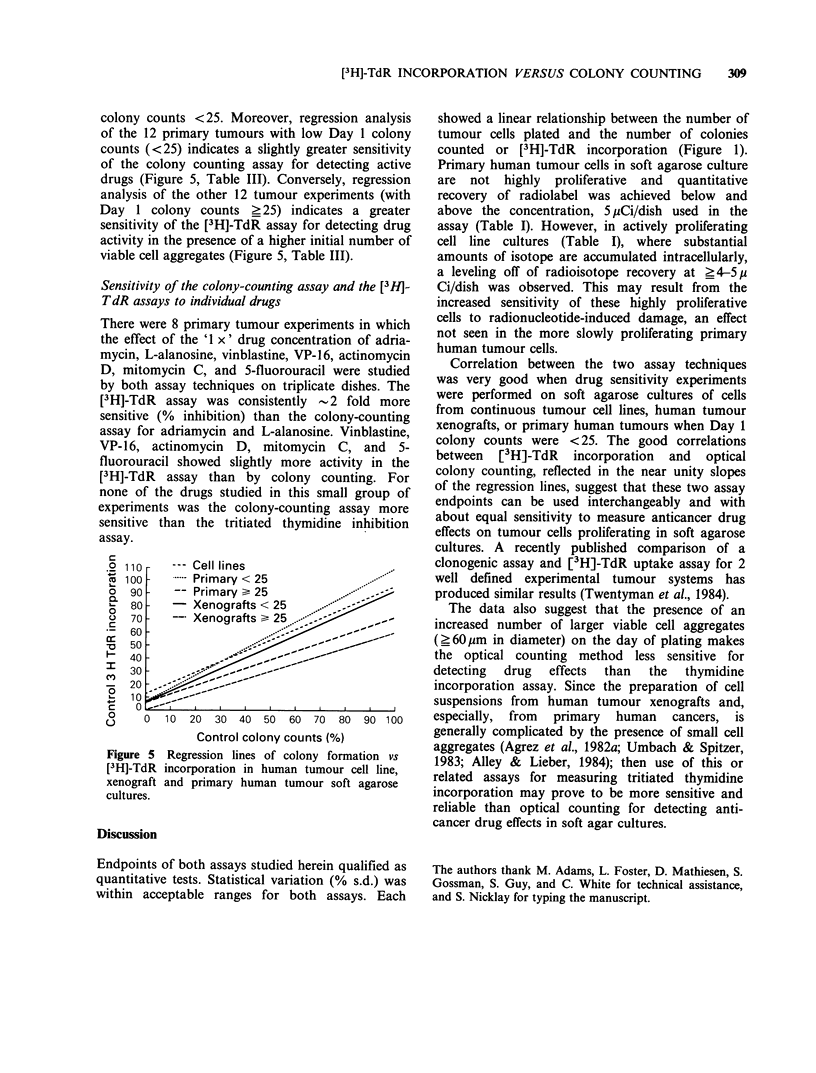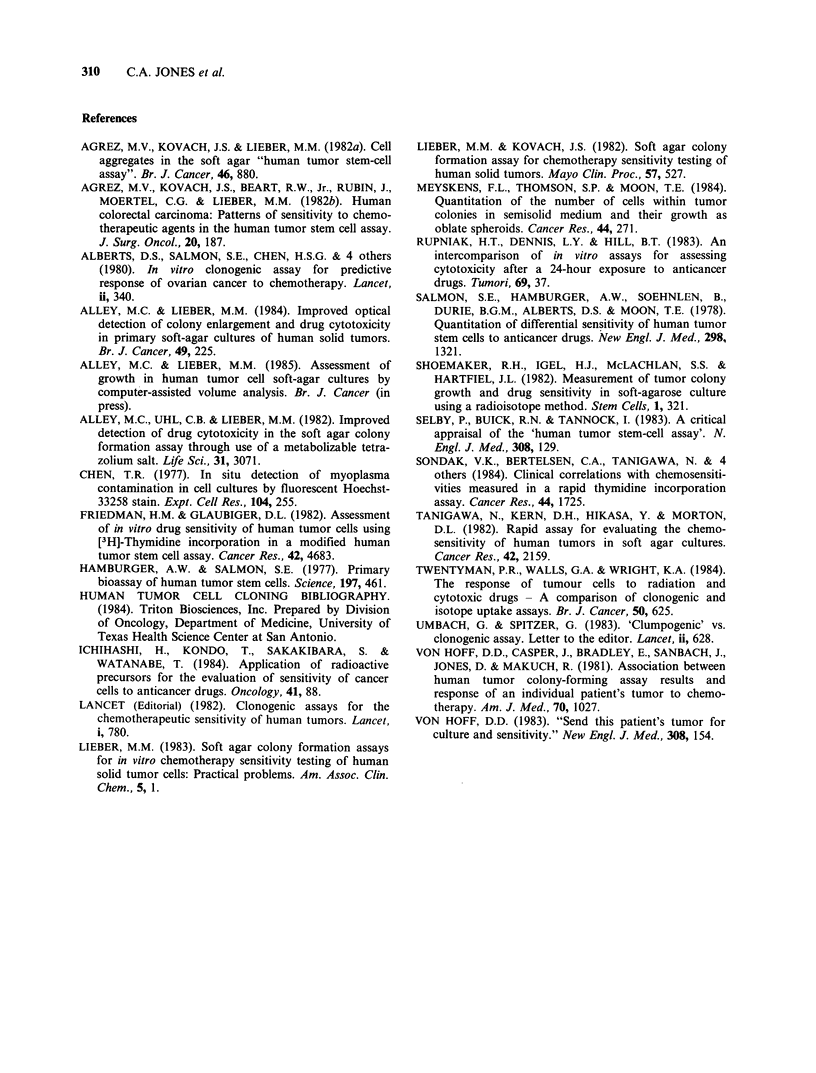# Soft agarose culture human tumour colony forming assay for drug sensitivity testing: [3H]-thymidine incorporation vs colony counting.

**DOI:** 10.1038/bjc.1985.194

**Published:** 1985-09

**Authors:** C. A. Jones, T. Tsukamoto, P. C. O'Brien, C. B. Uhl, M. C. Alley, M. M. Lieber

## Abstract

In vitro drug sensitivity testing, both by optical colony counting and by a [3H]-TdR incorporation assay, was performed on human tumour cells proliferating in soft agar cultures. Cells from two different human tumour cell lines, 5 different human tumour xenografts, and 94 different primary human tumour specimens of various histologic types were studied. Regression analysis comparing the results of the colony counting assay and the [3H]-TdR assay revealed good to excellent correlations between the two assay endpoints for quantitating the effect of in vitro anticancer drug exposure for a large number of different agents. The presence of pre-existing tumour cell aggregates complicates the performance of the optical colony counting assay. The [3H]-TdR incorporation assay is more sensitive and reproducible than the colony counting assay when performed on samples containing a large number of initially seeded tumour cell aggregates.


*Br. J. Cancer* (1985), **52**, 303–310

### Soft agarose culture human tumour colony forming assay for drug sensitivity testing: [$^3$H]-Thymidine incorporation *vs* colony counting

C.A. Jones[2], T. Tsukamoto[3], P.C. O'Brien[1], C.B. Uhl[1], M.C. Alley[4] & M.M. Lieber[1]

[1]*Mayo Clinic and Mayo Foundation, Rochester, Minnesota 55905, USA;* [2]*Department of Oncology Research, Health Services Center, University of Calgary, Calgary, Alberta, T2N 4N1 Canada;* [3]*Department of Urology, Sapporo Medical College Hospital, Sapporo 060, Japan; and* [4]*NCI Fredierick Cancer Research Facility PRI, Building 434, PO Box B, Frederick, MD 21401, USA.*

**Summary** *In vitro* drug sensitivity testing, both by optical colony counting and by a [$^3$H]-TdR incorporation assay, was performed on human tumour cells proliferating in soft agar cultures. Cells from two different human tumour cell lines, 5 different human tumour xenografts, and 94 different primary human tumour specimens of various histologic types were studied. Regression analysis comparing the results of the colony counting assay and the [$^3$H]-TdR assay revealed good to excellent correlations between the two assay endpoints for quantitating the effect of *in vitro* anticancer drug exposure for a large number of different agents. The presence of pre-existing tumour cell aggregates complicates the performance of the optical colony counting assay. The [$^3$H]-TdR incorporation assay is more sensitive and reproducible than the colony counting assay when performed on samples containing a large number of initially seeded tumour cell aggregates.

Use of short-term *in vitro* tumour colony forming assays (in agar, agarose, or methyl cellulose media) for assessing anticancer drug effects and for screening for new anticancer agents has been a popular area of oncologic research since the original publications by Salmon's group in 1977–1978 (Hamburger & Salmon, 1977; Salmon *et al.*, 1978). In the last seven years, more than 700 publications have described the general use of soft agar tumour colony forming assays for quantitation of cancer cell proliferation and anticancer drug effects *in vitro* (Human Tumor Cell Cloning Bibliography, 1984). Promising reports of good correlation between results of *in vitro* soft agar colony forming assays and patients' clinical response or resistance to chemotherapeutic agents have been published (Salmon *et al.*, 1978; Alberts *et al.*, 1980; Von Hoff *et al.*, 1981, 1983). Despite this extensive international experience, major questions remain about the biologic significance, technical performance and clinical utility of such assays (*Lancet* Editorial, 1982; Lieber & Kovach, 1982; Selby *et al.*, 1983; Agrez *et al.*, 1982*a*; Lieber, 1983).

The Mayo Clinic has had an intense interest in studying human tumour colony forming assays over the past 5 years. To date, this laboratory has studied over 6,200 different specimens of primary human cancers in soft agar colony formation assays similar to that described by Salmon and colleagues. This extensive experience has generated concern about technical problems which occur in the performance of soft agar colony forming assays using samples of cells isolated from primary human tumours; publications on this topic have appeared in this journal previously (Agrez *et al.*, 1982*a*; Alley & Lieber 1984, 1985). Consequently, it was of interest to investigate whether the use of a different way of assessing cell proliferation and drug effects in such soft agar tumour colony forming assays might be as effective or more effective than simply optically counting the number of colonies formed. Use of a thymidine incorporation measurement of cell proliferation applied to soft agar culture dishes appeared relevant because promising experience with such a method had been reported by several groups (Friedman and Glaubiger, 1982; Tanigawa *et al.*, 1982; Shoemaker *et al.*, 1982; Johnson and Glaubiger, 1983; Rupniak *et al.*, 1983; Sondak *et al.*, 1984; Ichihashi *et al.*, 1984).

The present manuscript reports experience in studying drug sensitivities of paired samples of human tumour cells, in a soft agarose colony forming assay, using both a standard computerized optical image analysis colony counting endpoint, and a thymidine incorporation endpoint patterned as closely as possible after the methodology described by Dr. Kern and his group (Tanigawa *et al.*, 1982).

Correspondence: M.M. Lieber
Received 22 April 1985.

© The Macmillan Press Ltd., 1985

### Materials and methods

### *Continuous human tumour cell lines*

Human tumour cell lines used were: SW-480 (metastatic colon carcinoma) (American Type Culture Collection) and A1663 (transitional cell carcinoma), laboratory of Dr. G. Todaro (Fredrick Cancer Research Center, Frederick, MD). All cell lines were mycoplasma-free (Fluorescence technique; HOECHST-33258; (Chen, 1977)) and grown as monolayer cultures in 75 $cm^2$ flasks (Falcon Plastics) in Dulbecco's Modified Eagle Medium [DMEM] supplemented with 10% calf serum, 2 mM L-glutamine, and 25 mM HEPES (Gibco Laboratories, NY). Cells in monolayer culture were dissociated in the presence of 0.1% trypsin (Gibco Laboratories, NY), washed once and resuspended in standard culture medium for immediate use or re-establishment of flask cultures.

### *Human tumour cell line soft agarose cultures*

All 35 mm culture dishes (Falcon Plastics) contained a 1 ml base layer of 0.5% Sea Plaque agarose (FMC Corporation) in fully supplemented DMEM media. Two human tumour cell lines, SW-480 and A1663, were plated out in soft agarose as follows: on Day 0, cells were harvested from monolayer culture, washed and resuspended at $2 \times 10^4$ cells ml in fully supplemented DMEM culture medium and molten 1.5% agarose (to a final concentration of 0.3%). This cellular suspension (0.5 ml) was applied to the base layer ($10^4$ cells/dish) and allowed to set at 4°C for 6 min. Duplicate soft agarose cultures were established for each experiment to assess drug dose responses by either colony formation or [$^3$H]-TdR incorporation. Drug or medium (in a 0.1 ml volume) was applied to the cellular layer of each culture dish, and cultures were placed in Wedco cell culture incubators at 37°C, 5% $CO_2$ and 100% relative humidity for 6 to 8 days.

### *Human tumour xenograft soft agarose cultures*

Eight experiments were performed on 5 different xenograft tumour specimens which included 1 ovarian and 4 different renal-cell carcinomas. Solid primary human tumour specimens were inoculated and serially passaged s.c. in male nude athymic 4-week-old BALB/c mice (Sprague-Dawley, Madison, Wisconsin). Xenograft tumours were excised and processed for growth assay using the same techniques as for primary tumour specimens.

### *Primary human tumour soft agarose cultures*

Freshly obtained human carcinoma specimens were dispersed enzymatically, filtered and placed into soft agarose cultures according to the method of Alley & Lieber (1984). Minced tumour tissue was disaggregated in RPMI 1640 medium containing 10% foetal calf serum, 0.6% collagenase II (Sigma Chemical Co., St. Louis, Missouri), and 0.002% DNase I (Sigma Chemical Co.) for 2 h, followed by filtration through a 48 $\mu$m (pore size) Nytex® nylon mesh (Tetko, Inc., Elmsford, NY). The cellular suspension was washed and diluted to 500,000 nucleated cells $ml^{-1}$ of fully supplemented CMRL 1066 medium and agarose (final concentration 0.3%) and 1 ml aliquots were applied to the culture base layer (500,000 cells/culture dish) and allowed to set at 4°C for 10 min. Duplicate soft agarose cultures were established for each primary tumour specimen to assess drug dose response by either colony counting or [$^3$H]-TdR incorporation. Drug or medium was applied to the cellular layer in a 0.1 ml volume just prior to placement of cultures into the Wedco cell culture incubators at 37°C, 5% $CO_2$ and 100% relative humidity for 10 to 14 days.

### *Reagents*

Stock anticancer drugs for this study were stored at −20°C. All drug dilutions were performed in unsupplemented Modified Eagle Medium (Gibco Laboratories, NY). Drugs were thawed, dilutions made, and application to cultures accomplished within 30 min. Drug was applied to cultures on the same day they were set up. A '1x' concentration of chemotherapeutic drug was designed to approximate the mean plasma concentrations estimated to be in patients' plasma 1 h after administration of a maximal clinically tolerable dose (Agrez *et al.*, 1982*b*). '1x' concentrations of the following drugs are (in $\mu g\,ml^{-1}$): actinomycin D (0.01), adriamycin (0.60), L-alanosine (50.0), bleomycin (2.0), 5-fluorouracil (10.0), methyl GAG (5.0), methotrexate (1.0), mitomycin C (0.40), cisplatinum (1.50), vinblastine (0.05), teniposide (VM-26) (10.0), etoposide (VP-16) (10.0). Drug dose response experiments were performed with 10×, 1×, and 0.1× concentrations of drug in primary human tumour and xenograft tumour experiments, and with 1×, 0.1×, 0.01×, 0.001× and 0.0001× concentrations in human tumour continuous cell line experiments. The control soft agarose cultures received 0.1 ml unsupplemented culture medium and the drug-treated cultures received 0.1 ml drug, both of which were applied on top of the cellular agarose layer. There were 6 dishes per control group and 3 dishes per drug-treated group (continuous drug exposure).

The vital stain, 2-(-iodophenyl)-3-(p-nitro-phenyl)-5-phenyl tetrazolium chloride (INT) was obtained from Aldrich Chemical Company. Stock INT at 1 mg $ml^{-1}$ in distilled water was prepared twice a week as described previously (Alley *et al.*, 1982).

Culture dishes from each group received 1 ml of stock INT at the indicated times and were re-incubated at 37°C for a further 24 h. The cultures were then stored at 4°C until colony formation was assessed by computerized image analysis (see below) within 48 h.

*Assessment of colony sizes and numbers*

The number and sizes of viable INT-stained colonies were determined on a 500 mm$^2$ area of each petri dish using a computerized image analyzer, FAS-II (Omnicon Feature Analysis System, Model II, Bausch & Lomb, Inc.). The Omnicon stem cell program computed the number of standard colonies for a 500 mm$^2$ area that had diameters of 30, 43, 60, 86, 122, or 173 $\mu$m. The mean viable colony count and standard deviation was recorded. A 60 $\mu$m D control (no drug treatment) colony is a 'standard' size used to measure colony formation in the soft agar colony forming assay for cell line, xenograft and primary tumour cells. (Agrez *et al.*, 1982*a*). The number of cells in such a 60 $\mu$m D colony is highly variable from tumour to tumour. (Meyskens *et al.*, 1984).

To determine the numbers of viable 60 $\mu$m cellular aggregates present at the time of drug application ('Day 1 count') in primary and xenograft tumour cultures, INT was applied to a set of triplicate control dishes from all xenograft and primary human tumour specimens immediately following placement into agarose culture. Primary tumour agarose cultures were considered acceptable if: The number of viable colony counts was $<25$ on Day 1, the number of viable 60 $\mu$m D images increased by at least $1\frac{1}{2}$ fold (minimum of 36) over that determined on Day 1 of incubation (Agrez *et al.*, 1982*a*), and the uniformly cytotoxic 'positive control drug' (100 $\mu$g ml$^{-1}$ mercuric chloride) produced a $\geqq 70\%$ decrease in 60 $\mu$m colony count.

*Assessment of [$^3$H]-thymidine incorporation*

[$^3$H]-TdR incorporation was determined according to a modification of the method of Tanigawa *et al.* (1982). Five $\mu$Ci [$^3$H]-TdR (2.0 Ci mmol$^{-1}$) (NEN, Boston, MA) was applied to each culture dish in 0.25 ml unsupplemented DMEM, 72 h after plating. The plates were incubated at 37°C an additional 24 h and [$^3$H]-TdR incorporation was terminated by placing the cultures at $-20$°C. Cultures were thawed and harvested as follows: The contents of each 35 mm$^2$ dish were transferred into a 15 ml polypropylene centrifuge tube (No. 25319, Corning Glassworks, Corning, New Jersey). Agarose was solubilized by addition of 10 ml of hot (96°C) magnesium-calcium-free PBS and the tubes were placed in a boiling water bath for 40 min. Tubes were then centrifuged at 2,500 r.p.m. for 15 min at room temperature. The supernatant was decanted and the pellet washed with 5 ml hot magnesium-calcium-free PBS. Following a further centrifugation at 2,500 r.p.m. for 10 min, the supernatant was aspirated and discarded. Five millilitres of ice cold 10% trichloroacetic acid (TCA) and 10 mg % human serum albumin were added and tubes were stored for 1 h at 4°C. The pellet was collected by centrifugation, washed once in 5% TCA and dissolved in 0.2 ml 2.0 N KOH for 1 h, then transferred to a scintillation vial containing 6 ml scintillation fluid (Safety Solve, Research Products International, Mount Prospect, IL). Radioactivity in each vial was determined by a Beckman LS7000 scintillation counter with a [$^3$H] counting efficiency of 57.73%. Background counts (c.p.m. of dishes labelled at $-20$°C, which was routinely $\leqq 80$ c.p.m.) are automatically subtracted from the control counts and the results are expressed as a percent of this 'corrected' control. [$^3$H]-TdR incorporation cultures were considered an acceptable assay if: (1) the number of c.p.m. in the control dishes was $\geqq 300$ c.p.m.; (2) the positive control drug (100 $\mu$g ml$^{-1}$ mercuric chloride) produced a $\geqq 90\%$ decrease in [$^3$H]-TdR incorporation from control values; (3) drug dose dependent changes in [$^3$H]-TdR incorporation were observed.

One culture was deemed evaluable with a control count $\geqq 260$ c.p.m. but that fulfilled the other two criteria.

*Statistical analysis*

The relationship between colony formation and [$^3$H]-TdR incorporation was described by linear regression analysis. Each cell line, xenograft and primary tumour experiment was analyzed separately, and the mean of the regression coefficients and correlations was obtained. Regression analysis was performed on a Hewlett Packard 9845-B computer. Paired data points were excluded from the analysis if either of the two values exceeded 150% of control (22 out of the total of 612 paired data points were excluded for this reason). Differences in correlations for two samples were based on a Student's *t*-test.

Nonlinear (quadratic) regression analysis was also performed on all the data. Although correlations were very slightly improved using this analysis, no meaningful difference was noted.

### Results

*Validity of the [$^3$H]-thymidine assay in soft agarose:*

1. *Number of cells plated and [$^3$H]-TdR incorporation.* There was a linear increase in [$^3$H]-TdR incorporation and colony count with increasing numbers of

**Figure 1** Number of nucleated primary human tumour cells plated *versus* [$^3$H]-TdR incorporation [●] (left side of figure) and colony counts [■] (right side). Tumour type: Primary human lung adenocarcinoma (Grade IV). Bars, ±s.d.

**Table I** [$^3$H]-Thymidine recovery in soft agarose cultures

| *Name* | *μCi[$^3$H] added* | *C.p.m. Recovered + s.d.* |
|---|---|---|
| Cell line | | |
| A1663 | 0.1 | 487.7±49.2 |
| | 1 | 2,952.7±219 |
| | 2 | 5,373±797 |
| | 4 | 7,360±1,286 |
| | 8 | 8,448.3±728 |
| 1° Human tumour | | |
| Uterus | 0.1 | 33±4 |
| | 1 | 74.7±7.2 |
| | 2 | 162.3±17 |
| | 4 | 257.3±76 |
| | 8 | 468.7±6 |

Increasing μCi quantities of [$^3$H]-TdR were applied to cultures 72h after plating. Cultures were re-incubated for 24h at 37°C and [$^3$H]-TdR incorporation terminated by placing at −20°C. Dishes were harvested as indicated in the **Materials and methods**.

lung adenocarcinoma cells plated (freshly excised primary human tumour) (Figure 1).

2. [$^3$H]-*Thymidine recovery from soft agarose cultures* A linear increase in radiolabel recovery was observed with cells from a uterine primary human tumour specimen (Table I). A linear increase followed by a leveling off at ≧4μCi added [$^3$H]-thymidine was observed with cells from the human transitional carcinoma continuous cell line A1663.

*Continuous tumour cell lines*

Cells from the human continuous cell lines A1663 and SW-480 proliferate well in soft agarose cultures and form large colonies when seeded at low concentration. Preparation of single cell suspensions from monolayer cultures is straightforward and true clonal growth of cells from these continuous cell lines is observed by microscope in soft agarose cultures. The plating efficiencies for forming 60μm diameter colonies for SW-480 and A1663 cells were 25% (±1.0%) and 15% (±1.2%), respectively, after 6 days in culture. A typical number of 30, 43, 86 and 122μm diameter colonies was 2,500, 2,100, 260, and 20 respectively, for SW-480 cells, and 850, 600, 43 and 2 respectively, for A1663 cells after 6 days in soft agarose culture. This represented median control colony counts of 1040 (range 546–1801) for SW-480 cells and 261 (range 100–696) for A1663 cells. A median control [$^3$H]-TdR incorporation of 49,749c.p.m. (range 42,095–54,856) and 15,012c.p.m. (range 13,044–36,411) was seen for SW-480 and A1663 cells, respectively. INT stained soft agarose cultures of A1663 and SW480, that had been previously incubated with 5μCi [$^3$H]-TdR for 24h, did not show a significant decline in viable colony count from untreated controls, indicating that the amount of added [$^3$H]-TdR was not cytotoxic. The correlation between control (non-drug treated) cultures assessed by optical colony counts or [$^3$H]-TdR incorporation, from all 6 experiments, was not strong (R: 0.66, See Table II). However, when drug tests (82 separate drug tests in 6 separate experiments) were performed on cells from the A1663 and SW-480 human tumour cell lines (Figure 5, Table III), the correlation was excellent between colony counts and [$^3$H]-TdR incorporation. *This excellent correlation was seen only when datum points within each individual experiment were compared.* An exemplary plot of data from one cell line experiment can be seen in Figure 2. The averaged regression line for all 6 experiments indicates that the optical/counting endpoint was slightly more sensitive than the [$^3$H]-TdR assay for detecting anticancer drug effects (Figure 2, Table III).

*Human tumour xenograft specimens*

A total of 8 separate experiments were performed on samples of 5 different human tumour xenograft specimens serially carried in nude-athymic mice. The human tumour xenograft specimens were prepared by enzymatic digestion and filtration in exactly the same manner as the primary human tumour samples described below. However, in general, cells from the xenograft tumours proliferated better *in vitro* than the primary human tumour samples but not as well as cells from the *in*

**Table II** Control [$^3$H]-TdR incorporation and optical colony counts in cell line soft agarose cultures

| *Cell line* | *Exp. No.* | *[$^3$H]-TdR incorporation c.p.m. ± s.d.* | *Mean optical colony count ± s.d.* |
|---|---|---|---|
| A1663 | 1 | 13,044.0 ± 2,556.7 | 75.3 ± 26.3 |
| | 2 | 36,410.0 ± 6,298.9 | 696.0 ± 60.0 |
| | 3 | 15,012.0 ± 1,651.3 | 261.0 ± 42.0 |
| SW-480 | 1 | 49,794.0 ± 3,585.0 | 1,801.0 ± 132.0 |
| | 2 | 42,095.0 ± 5,514.4 | 546.0 ± 67.0 |
| | 3 | 54,856.0 ± 8,392.9 | 1,038.0 ± 76.2 |

These control cultures were plated, incubated and endpoints assessed as indicated in the **Materials and methods**. The correlation (R) between control [$^3$H]-TdR incorporation and optical colony counting for these 6 experiments was 0.660.

**Table III** Linear regression analysis of optical colony counting and [$^3$H]-TdR incorporation[b]

| | *Slope* | *Intercept* | *R* | *No. of individual drug tests* | *No. of experiments* |
|---|---|---|---|---|---|
| Cell lines | 0.823 ± 0.099[a] | 12.201 ± 5.486 | 0.918 ± 0.043 | 83 | 6 |
| Xenografts | 0.860 ± 0.306 | 5.802 ± 3.416 | 0.859 ± 0.031 | 76 | 4 |
| Primary tumours | 1.021 ± 0.400 | 6.327 ± 8.577 | 0.845 ± 0.103 | 186 | 12 |

[a]All values ± s.d. [b]Day 1 counts < 25 (see **Results** section).

**Figure 2** Regression line of optical colony counts *vs* [$^3$H]-TdR incorporation. Human tumour cell line A1663. Each datum point (x) represents the paired % control value obtained by optical colony counting and [$^3$H]-TdR incorporation as indicated in the **Materials and methods**. Number of paired observations = 24. $R = 0.963$. (● = single datum point, ✱ = multiple data point.)

*vitro* continuous cell lines; the xenograft median control optical colony counts were 166 (range, 50–985) and the mean thymidine incorporation counts were 17,260 c.p.m. (range 2,349–140,251). (Plating efficiencies ranged from 0.01–0.20%.) The correlation between control colony counts and control [$^3$H]-TdR incorporation, taking values from all 8 experiments together, was poor.

When assay comparisons were carried out within an individual experiment, the correlation (Figure 5, Table III) between optical colony counts and thymidine counts was good. Assays for drug sensitivity (76 different drug tests) were compared in the 4 human tumour xenograft experiments in which Day 1 colony counts were < 25 colonies (median Day 1 counts of 4.5, range 0–9.5 [mean 4.6]). The averaged regression line for these four experiments indicates that there is no significant difference between the two assay endpoints for detecting antiproliferative drug activity for the drugs tested (Figure 5, Table III). An exemplary

**Figure 3** Regression line of optical colony counts *vs* [$^3$H]-TdR incorporation. Xenograft # RC-2 (Renal tumour). Each data point (x) represents the paired % control value obtained by optical colony counting and [$^3$H]-TdR incorporation. Number of paired observations = 12. R = 0.833. (● = single datum point, ✱ = multiple data point.)

plot of data from one xenograft experiment is presented in Figure 3. In contrast, correlation between assay methods was weaker in four xenograft experiments where Day 1 colony counts were ≧25 (median 117, range 51–305 [mean 147], 70 drug tests, Figure 5, Table IV). The averaged regression line for these experiments suggests a greater sensitivity for detecting drug activity using the thymidine assay (rather than optical colony counting) when an increased number of larger viable cell aggregates is present in the cultures when the drug is first applied.

*Primary human tumour specimens*

Ninety-four primary human carcinoma specimens of various histologic types were studied. Tumour types included: colon (22), kidney (13), endometrium (13), lung (10), ovary (9), breast (5), bladder (3), rectum (3), melanoma (3), omentum (3), small bowel (3), testis (2), brain (2), leiomyosarcoma (abdominal) (1), adrenal gland (1), and liver (1). For these 94 primary tumour experiments, only 17 (18%) were fully acceptable by standard colony counting criteria, whereas 56 of the 94 (60%) were evaluable by [$^3$H]-TdR assay criteria.

The plating efficiencies of the 24 primary tumours that were assessed by both colony counting and [$^3$H]-TdR incorporation ranged from 0.007–0.14%. Twelve of the primary human tumour cultures assayed by both methods were fully acceptable and thus had Day 1 colony counts of <25, and included tumour samples of 3 lung, 2 ovary, 2 colon, 2 omentum, 1 kidney, 1 melanoma, and 1 small bowel tumour. The mean Day 1 count for this group was 3.5 (range 0–11). An analysis of the regression lines for the 12 primary human tumour cultures with Day 1 colony counts <25 indicated a good correlation between optical colony counting and [$^3$H]-TdR incorporation when assessing drug effects (186 individual drug tests, Figure 5, Table III). A plot of data from one experiment can be seen in Figure 4.

**Figure 4** Regression line of optical colony counts *vs* [$^3$H]-TdR incorporation. Primary human tumour #5386 (colon tumour). Each datum point (x) represents the paired % control value obtained by optical colony counting and [$^3$H]-TdR incorporation. Number of paired observations = 18. R = 0.949. (● = single datum point, ✱ = multiple data point.)

Twelve other primary human tumour cultures were evaluated by both methods but had Day 1 colony counts ≧25. These 12 experiments provided 198 separate drug tests for which correlation analysis could be performed. The mean correlation for these 12 experiments was significantly decreased ($P$ = 0.015) (Table IV) when compared with the other 12 primary tumour experiments with Day 1

**Table IV** Linear regression analysis of optical colony counting and [$^3$H]-TdR incorporation[b]

| | *Slope* | *Intercept* | *R* | *No. of individual drug tests* | *No. of experiments* |
|---|---|---|---|---|---|
| Xenografts | 0.609 ± 0.321[a] | −0.506 ± 5.554 | 0.755 ± 0.144 | 70 | 4 |
| Primary tumours | 0.661 ± 0.204 | 5.598 ± 11.437 | 0.736 ± 0.099 | 198 | 12 |

[a]All values ± s.d. [b]Day 1 counts ≧ 25 (see **Results** section).

colony counts <25. Moreover, regression analysis of the 12 primary tumours with low Day 1 colony counts (<25) indicates a slightly greater sensitivity of the colony counting assay for detecting active drugs (Figure 5, Table III). Conversely, regression analysis of the other 12 tumour experiments (with Day 1 colony counts ≧25) indicates a greater sensitivity of the [³H]-TdR assay for detecting drug activity in the presence of a higher initial number of viable cell aggregates (Figure 5, Table III).

*Sensitivity of the colony-counting assay and the [³H]-TdR assays to individual drugs*

There were 8 primary tumour experiments in which the effect of the '1 ×' drug concentration of adriamycin, L-alanosine, vinblastine, VP-16, actinomycin D, mitomycin C, and 5-fluorouracil were studied by both assay techniques on triplicate dishes. The [³H]-TdR assay was consistently ~2 fold more sensitive (% inhibition) than the colony-counting assay for adriamycin and L-alanosine. Vinblastine, VP-16, actinomycin D, mitomycin C, and 5-fluorouracil showed slightly more activity in the [³H]-TdR assay than by colony counting. For none of the drugs studied in this small group of experiments was the colony-counting assay more sensitive than the tritiated thymidine inhibition assay.

**Figure 5** Regression lines of colony formation *vs* [³H]-TdR incorporation in human tumour cell line, xenograft and primary human tumour soft agarose cultures.

### Discussion

Endpoints of both assays studied herein qualified as quantitative tests. Statistical variation (% s.d.) was within acceptable ranges for both assays. Each showed a linear relationship between the number of tumour cells plated and the number of colonies counted or [³H]-TdR incorporation (Figure 1). Primary human tumour cells in soft agarose culture are not highly proliferative and quantitative recovery of radiolabel was achieved below and above the concentration, 5 μCi/dish used in the assay (Table I). However, in actively proliferating cell line cultures (Table I), where substantial amounts of isotope are accumulated intracellularly, a leveling off of radioisotope recovery at ≧4–5 μ Ci/dish was observed. This may result from the increased sensitivity of these highly proliferative cells to radionucleotide-induced damage, an effect not seen in the more slowly proliferating primary human tumour cells.

Correlation between the two assay techniques was very good when drug sensitivity experiments were performed on soft agarose cultures of cells from continuous tumour cell lines, human tumour xenografts, or primary human tumours when Day 1 colony counts were <25. The good correlations between [³H]-TdR incorporation and optical colony counting, reflected in the near unity slopes of the regression lines, suggest that these two assay endpoints can be used interchangeably and with about equal sensitivity to measure anticancer drug effects on tumour cells proliferating in soft agarose cultures. A recently published comparison of a clonogenic assay and [³H]-TdR uptake assay for 2 well defined experimental tumour systems has produced similar results (Twentyman *et al.*, 1984).

The data also suggest that the presence of an increased number of larger viable cell aggregates (≧60 μm in diameter) on the day of plating makes the optical counting method less sensitive for detecting drug effects than the thymidine incorporation assay. Since the preparation of cell suspensions from human tumour xenografts and, especially, from primary human cancers, is generally complicated by the presence of small cell aggregates (Agrez *et al.*, 1982*a*; Umbach & Spitzer, 1983; Alley & Lieber, 1984); then use of this or related assays for measuring tritiated thymidine incorporation may prove to be more sensitive and reliable than optical counting for detecting anticancer drug effects in soft agar cultures.

The authors thank M. Adams, L. Foster, D. Mathiesen, S. Gossman, S. Guy, and C. White for technical assistance, and S. Nicklay for typing the manuscript.

### References

AGREZ, M.V., KOVACH, J.S. & LIEBER, M.M. (1982*a*). Cell aggregates in the soft agar "human tumor stem-cell assay". *Br. J. Cancer*, **46**, 880.

AGREZ, M.V., KOVACH, J.S., BEART, R.W., Jr., RUBIN, J., MOERTEL, C.G. & LIEBER, M.M. (1982*b*). Human colorectal carcinoma: Patterns of sensitivity to chemotherapeutic agents in the human tumor stem cell assay. *J. Surg. Oncol.*, **20**, 187.

ALBERTS, D.S., SALMON, S.E., CHEN, H.S.G. & 4 others (1980). *In vitro* clonogenic assay for predictive response of ovarian cancer to chemotherapy. *Lancet*, **ii**, 340.

ALLEY, M.C. & LIEBER, M.M. (1984). Improved optical detection of colony enlargement and drug cytotoxicity in primary soft-agar cultures of human solid tumors. *Br. J. Cancer*, **49**, 225.

ALLEY, M.C. & LIEBER, M.M. (1985). Assessment of growth in human tumor cell soft-agar cultures by computer-assisted volume analysis. *Br. J. Cancer* (in press).

ALLEY, M.C., UHL, C.B. & LIEBER, M.M. (1982). Improved detection of drug cytotoxicity in the soft agar colony formation assay through use of a metabolizable tetrazolium salt. *Life Sci.*, **31**, 3071.

CHEN, T.R. (1977). In situ detection of myoplasma contamination in cell cultures by fluorescent Hoechst-33258 stain. *Expt. Cell Res.*, **104**, 255.

FRIEDMAN, H.M. & GLAUBIGER, D.L. (1982). Assessment of *in vitro* drug sensitivity of human tumor cells using [$^3$H]-Thymidine incorporation in a modified human tumor stem cell assay. *Cancer Res.*, **42**, 4683.

HAMBURGER, A.W. & SALMON, S.E. (1977). Primary bioassay of human tumor stem cells. *Science*, **197**, 461.

HUMAN TUMOR CELL CLONING BIBLIOGRAPHY. (1984). Triton Biosciences, Inc. Prepared by Division of Oncology, Department of Medicine, University of Texas Health Science Center at San Antonio.

ICHIHASHI, H., KONDO, T., SAKAKIBARA, S. & WATANABE, T. (1984). Application of radioactive precursors for the evaluation of sensitivity of cancer cells to anticancer drugs. *Oncology*, **41**, 88.

LANCET (Editorial) (1982). Clonogenic assays for the chemotherapeutic sensitivity of human tumors. *Lancet*, **i**, 780.

LIEBER, M.M. (1983). Soft agar colony formation assays for *in vitro* chemotherapy sensitivity testing of human solid tumor cells: Practical problems. *Am. Assoc. Clin. Chem.*, **5**, 1.

LIEBER, M.M. & KOVACH, J.S. (1982). Soft agar colony formation assay for chemotherapy sensitivity testing of human solid tumors. *Mayo Clin. Proc.*, **57**, 527.

MEYSKENS, F.L., THOMSON, S.P. & MOON, T.E. (1984). Quantitation of the number of cells within tumor colonies in semisolid medium and their growth as oblate spheroids. *Cancer Res.*, **44**, 271.

RUPNIAK, H.T., DENNIS, L.Y. & HILL, B.T. (1983). An intercomparison of *in vitro* assays for assessing cytotoxicity after a 24-hour exposure to anticancer drugs. *Tumori*, **69**, 37.

SALMON, S.E., HAMBURGER, A.W., SOEHNLEN, B., DURIE, B.G.M., ALBERTS, D.S. & MOON, T.E. (1978). Quantitation of differential sensitivity of human tumor stem cells to anticancer drugs. *New Engl. J. Med.*, **298**, 1321.

SHOEMAKER, R.H., IGEL, H.J., McLACHLAN, S.S. & HARTFIEL, J.L. (1982). Measurement of tumor colony growth and drug sensitivity in soft-agarose culture using a radioisotope method. *Stem Cells*, **1**, 321.

SELBY, P., BUICK, R.N. & TANNOCK, I. (1983). A critical appraisal of the 'human tumor stem-cell assay'. *N. Engl. J. Med.*, **308**, 129.

SONDAK, V.K., BERTELSEN, C.A., TANIGAWA, N. & 4 others (1984). Clinical correlations with chemosensitivities measured in a rapid thymidine incorporation assay. *Cancer Res.*, **44**, 1725.

TANIGAWA, N., KERN, D.H., HIKASA, Y. & MORTON, D.L. (1982). Rapid assay for evaluating the chemosensitivity of human tumors in soft agar cultures. *Cancer Res.*, **42**, 2159.

TWENTYMAN, P.R., WALLS, G.A. & WRIGHT, K.A. (1984). The response of tumour cells to radiation and cytotoxic drugs – A comparison of clonogenic and isotope uptake assays. *Br. J. Cancer*, **50**, 625.

UMBACH, G. & SPITZER, G. (1983). 'Clumpogenic' vs. clonogenic assay. Letter to the editor. *Lancet*, **ii**, 628.

VON HOFF, D.D., CASPER, J., BRADLEY, E., SANBACH, J., JONES, D. & MAKUCH, R. (1981). Association between human tumor colony-forming assay results and response of an individual patient's tumor to chemotherapy. *Am. J. Med.*, **70**, 1027.

VON HOFF, D.D. (1983). "Send this patient's tumor for culture and sensitivity." *New Engl. J. Med.*, **308**, 154.